# Tracking perceptual decision mechanisms through changes in interhemispheric functional connectivity in human visual cortex

**DOI:** 10.1038/s41598-018-37822-x

**Published:** 2019-02-04

**Authors:** Teresa Sousa, João V. Duarte, Gabriel N. Costa, Valentin G. Kemper, Ricardo Martins, Rainer Goebel, Miguel Castelo-Branco

**Affiliations:** 1 Coimbra Institute for Biomedical Imaging and Translational Research (CIBIT), University of Coimbra, Coimbra, Portugal; 2 Institute of Nuclear Sciences Applied to Health (ICNAS), University of Coimbra, Coimbra, Portugal; 3 Institute for Biomedical Imaging and Life Sciences (CNC.IBILI), Faculty of Medicine, University of Coimbra, Coimbra, Portugal; 4 Department of Cognitive Neuroscience, Faculty of Psychology and Neuroscience, University of Maastricht, Maastricht, Netherlands; 5 Department of Neuroimaging and Neuromodeling, Netherlands Institute for Neuroscience, Royal Netherlands Academy of Arts and Sciences (KNAW), Amsterdam, Netherlands

## Abstract

The role of long-range integration mechanisms underlying visual perceptual binding and their link to interhemispheric functional connectivity, as measured by fMRI, remains elusive. Only inferences on anatomical organization from resting state data paradigms not requiring coherent binding have been achieved. Here, we used a paradigm that allowed us to study such relation between perceptual interpretation and functional connectivity under bistable interhemispheric binding vs. non-binding of visual surfaces. Binding occurs by long-range perceptual integration of motion into a single object across hemifields and non-binding reflects opponent segregation of distinct moving surfaces into each hemifield. We hypothesized that perceptual integration vs. segregation of surface motion, which is achieved in visual area hMT+, is modulated by changes in interhemispheric connectivity in this region. Using 7T fMRI, we found that perceptual long-range integration of bistable motion can be tracked by changes in interhemispheric functional connectivity between left/right hMT+. Increased connectivity was tightly related with long-range perceptual integration. Our results indicate that hMT+ interhemispheric functional connectivity reflects perceptual decision, suggesting its pivotal role on long-range disambiguation of bistable physically constant surface motion. We reveal for the first time, at the scale of fMRI, a relation between interhemispheric functional connectivity and decision based perceptual binding.

## Introduction

The visual system segments and binds local stimulus’ features to produce globally coherent percepts. The question of how this system binds signals coming from distributed contours into single or multiple objects or surfaces, is known as the binding problem^1,2^. In the motion domain, this requires the knowledge of how the visual system integrates global patterns of motion from its components. In particular, the physiological mechanisms underlying the integration of such elements across large portions of the visual field are known as long-range integration mechanisms^3^. Here, we investigated these mechanisms by studying perceptual interpretation of a bistable moving stimulus where coherence requires interhemispheric binding. It provides a challenge to long-range integration in perceptual decision under ambiguous conditions^4,5^.

The human motion complex (hMT+) is well known to be involved in motion perception^6,7^ and to contribute to the processing of simple bilateral visual stimuli such as flickering checkerboards^8^. Bistable apparent motion is also processed in this region, as shown by studies using the motion quartet paradigm, which leads to perception of either horizontal (across visual hemifields) or vertical (within one visual hemifield) motion^9–12^. Electroencephalographic (EEG) data have suggested stronger oscillatory coupling between right and left visual cortices during perception of horizontal motion compared with vertical motion of the motion quartet^11^, although it is difficult with this technique to ascribe a role to particular brain regions such as hMT+. These findings are consistent with the idea that in the case of perceived horizontal movement, information from regions in both visual hemispheres may show larger coupling.

In any case, evidence from functional connectivity data that an early visual area such as hMT+ can be pivotal in perceptual binding across hemispheres has so far been absent. If this hypothesis holds true, it would also inform the independent debate concerning bottom-up vs. top-down mechanisms in perceptual decision^13^. Moreover, structural connectivity data from callosal segments connecting hMT+ show a correlation with perceived horizontal apparent motion^12^. Nevertheless, the link between long-range perceptual mechanisms and functional connectivity remains unexplored, and only inferences on anatomical organization from resting state data have so far been attempted^14^. This motivated our current approach to test whether functional connectivity indexes decision-making at the early visual level.

We have shown, using plaid stimuli, that hMT+ underlies the perceptual binding of moving surfaces^15^. As plaids’ components overlap, these stimuli mainly recruit mechanisms that require local integration of motion in relatively small receptive fields. Thus, they cannot be used to study interhemispheric binding of visual motion features at a larger spatial scale. The fundamental novelty added by the stimulus paradigm used in the current study is the need for interhemispheric integration. We used a previously described bistable non-overlapping moving stimulus^16,17^ composed of a 1D component moving in each visual hemifield, which requires interhemispheric integration (1D components are interhemispherically integrated into a single 2D surface comprehending both hemifields) or segmentation (1D components are perceptually parsed into different objects). This allows us to specifically investigate the role of the hMT+ interhemispheric functional connectivity in the modulation of surface segregation vs. integration as a probe to investigate the role long-range interactions in decision. In a previous study we observed that long-range binding of this bistable stimulus mainly reflect bottom-up processes arising within hMT+^13^. As a step further, we set to investigate with high-resolution (7T) functional magnetic resonance imaging (fMRI), the role of interhemispheric functional connectivity across hMT+ in both hemispheres as a modulator of perceptual integration vs. segregation of multiple physically constant moving surfaces separated across hemifields.

To take advantage of the high-dimensional nature of 7T fMRI data we acquired a slab of data focused on hMT+ (although covering also other visual areas). This a priori choice of our region of interest was based on our own previous findings using fMRI data^13^, suggesting a role for this complex and heterogeneous region (containing MT proper and MST) in perceptual disambiguation of moving surfaces. Previously, we have found that hMT+ has a bottom-up dominant role during long-range binding and is critical for the maintenance of the perceptual representations (other areas were only involved during the short switch events)^13,15^. Furthermore, previous studies using EEG and structural MRI data had proposed that perceptual decision can be indexed in interhemispheric hMT+ communication^11,12^, reinforcing our a priori hypothesis. hMT+ contains “component” neurons responding to the 1-D motion and also neurons showing pattern selectivity, meaning that these neurons respond specifically to motion of more complex 2-D objects as a whole (“pattern” neurons), which is the case of our stimulus. There is single-cell electrophysiological evidence that neuronal computation of global 2-D or pattern motion is performed by such pattern selective neurons in hMT+, while neurons in early visual cortex, such as V1 and V2, are only sensitive to 1-D motion features of an object (virtually 100% of component motion neurons in these regions)^18,19^. It is therefore not expected that V1 and V2 differentially respond to stimuli requiring perceptual integration, as we have verified in our previous studies^13,15^.

Here, we aim to verify if the interhemispheric binding of visual motion features can be tracked through interhemispheric connectivity of hMT+. If perceptual output is associated with differential interhemispheric connectivity of this region, it indicates that hMT+ is involved in long-range perceptual binding.

## Results

### Behavioral analysis

All participants showed a sufficiently large number of perceptual switches for an event-related analysis, the same holding true, with the exception of one, for blocks of stable perception, which were balanced for each percept type (coherent and incoherent). During ambiguous stimulation, participants perceived coherent motion for 8.35 ± 0.96 seconds, on average, while the mean duration of the incoherent motion percept was 7.25 ± 0.70 seconds. On average each participant experienced 122.4 ± 15.4 perceptual switches. The gamma and lognormal distributions fit well each percept duration histogram, as revealed by the Kolmogorov-Smirnov test group results. The test demonstrated no significant deviation, at *P* = 0.05, between the fitted distributions and each percept duration histogram. Additionally, both distributions fitted similarly the data and both percepts duration histograms.

### hMT+ localization

**Figure 1:**
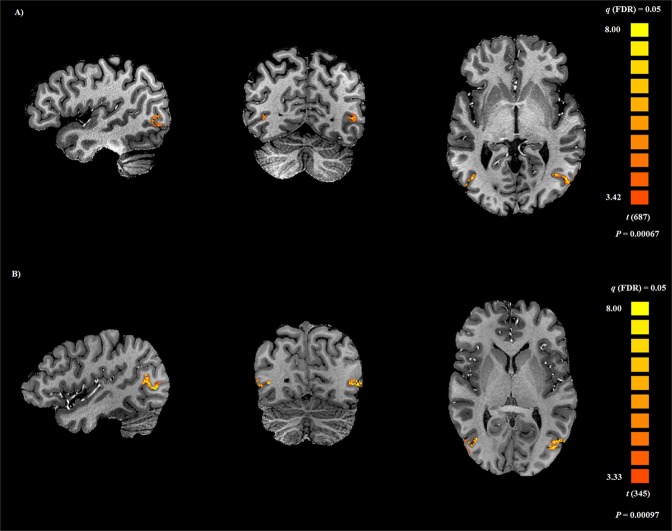
Right and left hMT+ localization in two participants (**A**,**B**). Activation maps resulting from the contrast between motion conditions and the static pattern condition during the localizer experiment. The hMT+ left and right are shown at the same statistical threshold level (*q*(FDR corrected) = 0.05).

Localization of hMT+ was the first imaging analysis stage and the basis of all subsequent steps. The contrast between the evoked brain activity during the localizer motion and no motion conditions produced statistical maps with clear bilateral activations (Fig. 1 shows the example from two participants) within the acquired slab (see the functional coverage in Supplementary Fig. S1). The defined regions-of-interest (ROIs) per participant included on average 512.89 ± 84.29 voxels from the right hemisphere and 811.33 ± 102.45 voxels from the left hemisphere in the expected spatial location of hMT+.

### Interhemispheric functional connectivity

**Figure 2:**
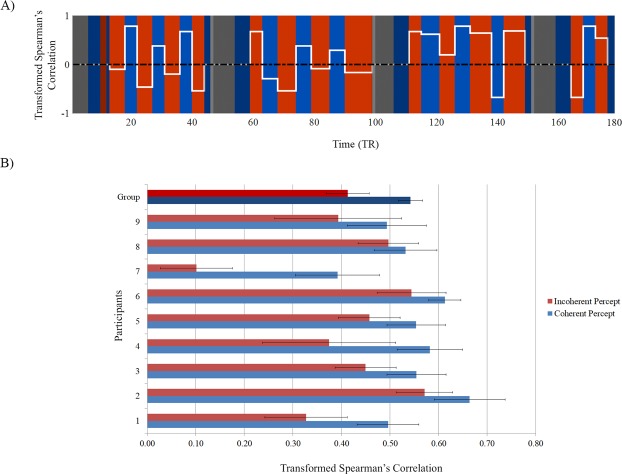
Interhemispheric functional connectivity per participant based on the mean of transformed partial correlation between activity modulations in left and right hMT+ during each type of motion percept. (**A**) Example of correlation estimation (white line) for one ambiguous run of one participant using hemodynamic delay of 3 TR. The red blocks represent the incoherent motion percept intervals and the blue blocks represent the coherent motion percept intervals. No motion condition intervals (gray blocks), perceptual intervals immediately after no motion condition and intervals with less than 4 TR were not included in the analysis (indicated with darkened areas). (**B**) Mean interhemispheric correlation values during each percept for all participants. The correlation values are shown with the standard error of the mean. Group differences were significant at *P* = 0.008.

In order to study the effect of the interhemispheric integration (binding) vs. segregation (non-binding across hemispheres) of the ambiguous moving stimulus, we calculated the interhemispheric partial correlation between activity patterns in the right and left hMT+ during coherent (bound) and incoherent (unbound) motion percepts. Figure 2A shows the mean transformed Spearman correlation between right and left hMT+ during a typical run of the ambiguous stimulation. Unlike what is seen in which concerns activation *beta* values (1.41 ± 0.21 during the coherent motion percept and 1.75 ± 0.23 during the incoherent motion percept, see Supplementary Fig. S2), correlation was higher during blocks of coherent motion percept than during blocks of incoherent motion percept in all participants (Fig. 2B). The interhemispheric hMT+ correlation was on average 0.54 ± 0.02 during the coherent motion percept and 0.41 ± 0.04 during the incoherent motion percept. This difference in interhemispheric hMT+ correlation between percepts is highly significant (*P* = 0.008, two-tailed paired sample Wilcoxon signed-rank test) and was not present for visual areas such as V1/V2. The control experiment revealed no significant differences between the hMT+ interhemispheric correlation during passive perceptual viewing of vertical (0.57 ± 0.04) and horizontal (0.57 ± 0.04) motion (*P* = 0.859, two-tailed paired sample Wilcoxon signed-rank test).

The interhemispheric functional connectivity variations depending on the visual motion percept were also estimated with a psychophysiological interaction (PPI) analysis, as the difference between the magnitude of the interaction regressors of the two perceptual states, when using one hMT+ as seed and analyzing the effect in the opposed one. The *beta* value of the contrast between the PPI predictors was on average higher during the coherent motion percept than during the incoherent motion percept for all participants (0.12 ± 0.03), which was significant at the group level as revealed by a 2-tailed binomial test (*P* = 0.004).

**Figure 3:**
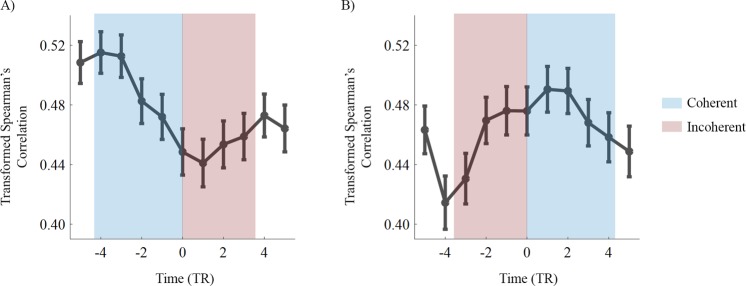
Group analysis of the interhemispheric hMT+ time varying correlation during the perceptual switches. Note the distinct trajectory and slopes in the correlation curve depending on whether participants experienced the perceptual switch from coherent to incoherent motion (**A**) or from incoherent to coherent motion (**B**). Correlation significantly decreases from coherent (bound) to incoherent (unbound) (*P* = 0.0005) and the opposite occurs from unbound to bound (*P* = 0.005). The blue and red blocks width shows the mean duration of coherent and incoherent motion percepts, respectively. The correlation values are presented after Fisher-z transform with the standard error of the mean and taking in account the hemodynamic delay of 3 TR.

As an additional test to the hypothesis that interhemispheric correlations at the level of hMT+ are tightly related to perception, we also investigated how the dynamics of interhemispheric correlation in time is associated with events of perceptual switches. A sliding window was used to trace an event related time course of the interhemispheric correlation before and after the perceptual switches in both directions, from coherent to incoherent motion (Fig. 3A) and vice-versa (Fig. 3B). We found opposite patterns of correlation changes associated with the perceptual alternation events. As expected from bilaterally represented homologous motion-sensitive brain regions, the baseline correlation between right and left hMT+ was large during visualization of bistable motion. Importantly, it decreased (from a maximum to a minimum value) around the perceptual switch from coherent to incoherent motion (see Fig. 3A) and increased (from a minimum to a maximum value) around the perceptual switch from incoherent to coherent motion (Fig. 3B).

A linear regression to the slope of each correlation curve (from −4 TR to 1 TR, extreme values of correlation within mean duration of percepts) established that there is a significant relationship between the dynamics of perceptual switches events and interhemispheric functional connectivity. We found that the slope is significantly different from zero when participants experienced the perceptual switches both from coherent to incoherent motion (*R*^2^ = 0.95; *F* (1, 5) = 102.03, *P* = 0.0005) and from incoherent to coherent motion (*R*^2^ = 0.86; *F* (1, 5) = 30.87, *P* = 0.005).

## Discussion

Here, we tested whether interhemispheric functional connectivity in visual area hMT+ reflects long-range perceptual binding and decision. We investigated this question by studying perceptual long-range integration vs. segregation of bistable visual moving surfaces. Importantly, we investigated the association between the dynamics of interhemispheric functional connectivity and perceptual switches to directly test our hypothesis. In other words, we asked whether interhemispheric binding (coherent percept) leads to increased functional connectivity and whether perceptual segregation (incoherent percept) of visual motion surfaces leads to decreased correlation patterns between homologous hMT+ regions across hemispheres.

Previous studies investigating neural responses in hMT+ to superimposed moving gratings, the so-called plaids^15^, did not allow for the study of interhemispheric binding of visual motion features. Furthermore, previous structural or fMRI studies on the role of visual regions in processing non-local patterns, only addressed the relation between structural connectivity and perception of apparent motion^12^, or the responses to bilateral vs. unilateral checkerboard flickering stimuli^8^. The former showed that structural connectivity in the corpus callosum was predictive of the perceptual bias to perceive horizontal apparent motion. The latter suggested that the lateral occipito-temporal regions and the medial occipital cortex may contribute to bilateral visual integration during early visual processing. However, none of them addressed the dynamics of interhemispheric functional connectivity across early visual areas, such as hMT+, in relation to long-range binding and perceptual decision. Using an apparent motion paradigm, an earlier and inspiring EEG study^11^, suggested that path integration in apparent motion, is reflected in the coupling between right and left visual cortices although its relation to hMT+ could not be demonstrated.

We were able to investigate this issue by studying bistability of truly moving surfaces involving integration across hemispheres. The fact that our physically constant moving stimulus required long-range integration across the vertical meridian, provides ideal conditions for studying interhemispheric communication^13,20^ and integration of motion from widely sampled component motion signals. The stimulus could be perceived as a coherent pattern comprehending both visual hemifields or as two separate (unbound/incoherent) patterns with distinct motion (i.e. perceived as moving in opposing directions, one in each visual hemifield). Our recent fMRI study at 3 Tesla^13^ investigated bottom-up and top-down mechanisms in decision with this stimulus, but did not explore fluctuations in correlated activity of bilateral homologous hMT+ regions during distinct perceptual transitions, as studied here at 7 Tesla. The results of that study revealed no involvement of the early visual cortex in the disambiguation of interhemispheric bistable moving surfaces. Here, we also did not find differential response of other visual regions than hMT+ related to the perceptual content. However, we cannot exclude that other areas play a role in this process. For example, we have previously found evidence that higher-order areas in the parietal cortex, are not related to percept maintenance but instead to fast perceptual switches^13^.

Functional connectivity analysis is known as an estimate of the neuronal activity interactions between two or more brain regions^21^. It is believed that functional connectivity based on fMRI signals reflects the ongoing neuronal activity coupling^22^. Simultaneous EEG-fMRI data have also demonstrated that BOLD responses during perceptual decision-making reflect electrophysiological responses in particular in which concerns brain oscillations^23^. Here, we studied the correlation of activity patterns between right and left hMT+. Different stimulation trials were averaged in order to increase the detection probability of functional connectivity variations^24^. The modulation of interhemispheric correlations as a function of the perceptual state supports our hypothesis that increased hMT+ interhemispheric functional connectivity might encode binding of motion information arising from motion signals sampled across large distances. The higher correlation values and psychophysiological interactions observed during the coherent (bound) percept, as compared to the incoherent (unbound) percept, mirrors the interhemispheric integration vs. segregation of visual information manifested during each perceptual experience. Furthermore, the fact that variations in correlation patterns signaled the timing of perceptual reversals corroborates the hypothesis that interhemispheric communication between visual areas, here studied using the dynamics of functional connectivity, serves as a binding mechanism. Importantly, based on a control experiment, we show that the interhemispheric correlation differences found during the ambiguous stimulation are due to perceptual binding and not due to mere perception of motion direction. These data provide evidence that hMT+ yields mechanisms for perceptual grouping operations underlying the switches between long-range integration and segregation of visual motion. Although the effects sizes of our results are not large (possibly because of the already large baseline correlation), they are quite significant. An outstanding question relates to the relative contribution of regions such as MST and FST, which also belong to the MT + complex, to interhemispheric connectivity. Here, we used a functional localizer approach, based on linear axis of motion mapping, that was previously shown to be mainly confined to MT proper^25^. We do therefore expect that the observed effect is largely dominated by a contribution of MT. Future studies, focusing on the heterogeneity of hMT+ clusters, should try to address into which extent MST and FST contribute to interhemispheric connectivity patterns.

We demonstrated for the first time evidence based on fMRI data of a close relation between interhemispheric functional connectivity and long-range perceptual integration. Accordingly, we found that the dynamics of the functional connectivity reflects the perceptual binding vs. perceptual segregation decisions leading to bound and unbound configurations of the bistable stimulus. It remains an interesting question what is exactly the neural mechanism behind the change in interhemispheric connectivity related to perceptual switches. It might reflect the synchronization of slow fluctuations in the underlying signals, which may be different between perceptual conditions. We add to previous suggestions that dynamic fluctuations in brain connectivity^26,27^ and oscillations^23^ relate to ongoing cognitive function. Our results are novel because in previous studies changing the task or the stimulus did not significantly alter connectivity much beyond observed resting state connectivity^28^. Resting state connectivity in hMT+ has been already explicitly tested in a recent fMRI study^14^. The authors used functional connectivity to investigate whether resting-state activity patterns map onto the anatomical and functional architecture of the brain. Visual tasks, such as apparent motion, were also applied but no evidence for perceptual modulation was identified using such path integration paradigm. Thus, our study is intriguing because it shows for the first time that it is possible to identify changes in interhemispheric connectivity related to perceptual decision. We believe that this may be related to the unique nature of our task involving changes in conscious perception and large-range integration of whole hemifield representations.

In conclusion, our results indicate that hMT+ interhemispheric functional connectivity modulation reflects perceptual switches involving differential long-range integration/segregation of visual moving stimuli, suggesting a crucial role for hMT+ on long-range perceptual binding and decision-making. The interhemispheric correlation changes across hMT+ depended on whether participants integrated all motion features into the percept of a single coherent pattern (bound configuration) or whether they segregated visual motion features (unbound). Future studies should elucidate the role, at finer temporal and spatial scales, of functional connectivity in long-range perceptual integration and binding.

## Methods

### Participants

Ten participants (six males; 28.7 ± 7.6 years; 9 right-handed) with normal or corrected-to-normal vision and with no history of psychiatric or neurological disorders participated in this study. Participants gave informed consent and were paid for their participation. All experimental procedures were conducted with approval from the Ethical Committees of Coimbra University and the Faculty of Psychology and Neuroscience of Maastricht University. The study has been conducted according to the principles expressed in the Declaration of Helsinki.

### Experimental design

We acquired a standard structural MRI sequence and fMRI data (an hMT+ functional localizer and ambiguous visual motion stimulation runs) from all participants. Furthermore, the participants performed familiarization sessions outside the scanner (two runs of ambiguous stimulation per day during the week before of the scanning session).

The familiarization sessions helped participants get used to the ambiguous stimulus and allowed us to verify whether robust bistability was present. Only participants with minimum average duration of six seconds per each motion percept were eligible for the imaging study.

The stimuli were created with MATLAB (version 2016a; The Mathworks, Inc.), using the Psychophysics Toolbox^29,30^. In the scanner, they were projected on a screen located at 99 cm away from the participant (screen size: 17.2° × 10.4° (horizontal × vertical); stimuli size: 11° × 10°; projection display: resolution of 1920 × 1080 and refresh rate of 60 Hz). Responses were collected through a magnetic resonance compatible button box (Current Designs, 4-button response device, Philadelphia, USA).

The structural and functional data were acquired with a Siemens MAGNETOM 7T scanner (Siemens; Erlangen, Germany) and a 32-channel head-coil (Nova Medical Inc.; Wilmington, MA, USA) at the facilities of the Faculty of Psychology and Neuroscience of Maastricht University. The pre-processing and analyses of the imaging data were performed using BrainVoyager QX (version 2.8.4; Brain Innovation, Maastricht, The Netherlands)^31^ and MATLAB. Statistical analyses were performed with the IBM (Armank, 169 NY) SPSS Statistics 22.0 software package.

### hMT+ Functional localizer

To functionally localize the hMT+ complex we showed participants moving gratings in multiple axes of motion. The stimulus was composed by 8 motion conditions (both directions of motion along axis of 0°, 45°, 90° and 135°) randomly interleaved with a no motion condition, all with the duration of 8 seconds. Each motion condition was repeated 9 times across 3 stimulation runs with 196 volumes each. All the motion conditions consisted of black moving lines on a white background (contrast: 100%; motion speed: 3°/s; duty cycle: 6%; spatial frequency: 0.6 cycle/°; stimulus visual angle: 11° × 10° – horizontal × vertical). A central blue cross (visual angle: 0.2°) was presented as a fixation target at the visual midline. Our axis of motion localizer approach took into account our previous finding that axes of motion mapping yield direction selective maps that are largely confined to the MT proper portion of hMT+, defined retinotopically^25^. In this way, in spite of not providing an explicit separation of MST from MT, our localizer strategy allows us to have a dominant contribution of MT proper to the defined ROIs.

The right and left hMT+ were defined per participant as the voxels in the middle temporal region responding significantly, at *q* (FDR) = 0.05, to the contrast of motion resulting from fixed effects general linear model (GLM) analyses.

### Ambiguous stimulus

**Figure 4:**
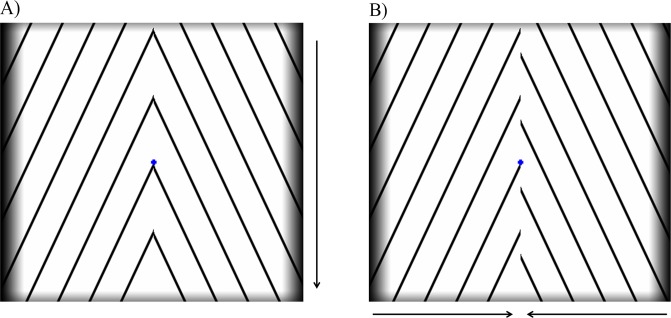
Ambiguous moving stimulus based on roof-shaped lines, which elicits two alternative perceptual interpretations. This paradigm allows us to study bistable perceptual transitions between interhemispheric long-range integration (**A** – the 1D components are integrated into a single 2D moving pattern comprehending both hemifields, coherent motion) and segregation (**B** – the 1D components are perceptually parsed into different objects, one in each hemifield, incoherent motion) of visual motion features. Arrows depict the direction of perceived motion. The amplified difference in phase in **B** represents only a perceptual feature of the illusion, as the physical stimulus was constant.

The ambiguous stimulus consisted of a roof-like pattern (oblique black lines equally separated forming an inverted V-shape on a white background) continuously moving downward (orientation: ± 25° relative to x-axis; contrast: 100%; motion speed: 3°/s; duty cycle: 6%; spatial frequency: 0.6 cycle/°; stimulus visual angle: 11° × 10° - horizontal × vertical) with a central blue fixation cross (visual angle: 0.2°). The design of the stimulus is based on the original description of Wallach^16,17^ and its direction of motion, parallel to vertical meridian, was chosen to challenge the interhemispheric coherence (so that incoherent motion did not cross the vertical meridian, unlike coherent motion, which required across meridian binding). In spite of the absence of physical change in the moving stimulus, participants spontaneously experienced perceptual alternations between coherent motion (the lines were coherently perceived as moving downward, as a single roof-like surface covering both hemifields – Fig. 4A) and incoherent motion (the lines were perceived as two separate surfaces, one in each visual hemifield, moving inward – Fig. 4B), reflecting its bistability. Participants reported the perceived motion pattern through a button press.

In order to optimize the stimulus for the presence of more stable percepts, it was prepared with a slight offset of 0.06° between the two 1D components and the line terminations on the stimulus border were smoothed using a mask with a central aperture (9.9° × 10°) superimposed on the roof-like surface^32^. The mask was prepared using a bi-dimensional squared Gaussian kernel (width and height: 0.6° ± 0.3°). The ambiguous stimulation was divided in four separate runs with 180 volumes each, completing 20 trials of motion (60 seconds each) interleaved with no motion periods (static figure of the roof-shaped stimulus presented for 15 seconds).

### Imaging data acquisition

Structural images were acquired for anatomical reference using a T1-weighted magnetization prepared rapid acquisition gradient echo (3D-MPRAGE) (256 sagittal slices; isotropic resolution of 0.6 mm; repetition time (TR) = 3100 ms; echo time (TE) = 2.52 ms; flip angle = 5°; matrix = 384 × 384). To correct for intensity inhomogeneities, additional gradient echo proton-density (GE-PD) images (same parameters as 3D-MPRAGE, except TR = 1440 ms) were acquired.

Functional images were obtained using gradient echo (T2-weighted) echo-planar imaging (2D GE-EPI) (28 slices; isotropic resolution of 0.8 mm; TR = 2000 ms; TE = 25.6 ms; flip angle = 69°; matrix = 186 × 186). After the GE-PD images, additional five functional volumes, with a reversed encoding direction, were acquired to correct for echo-planar imaging (EPI) distortions. Moreover, in order to ensure that functional images with isotropic resolution of 0.8 mm would be in the correct position to cover the bilateral ROI, a slice positioning functional run, with a protocol based on previous studies^25,33,34^, was first acquired (39 coronal slices; isotropic resolution of 1.6 mm; TR = 2000 ms; TE = 17.2 ms; flip angle = 70°; matrix = 88 × 88).

### Behavioral Data Analysis

In order to understand the dynamics of bistable perception, the mean duration of each percept and the number of perceptual switches per participant were estimated. The gamma and the lognormal distributions^4,35,36^ were fitted to the data using the maximum likelihood method to estimate the parameters per participant (time-window from zero to the longest percept duration) and the goodness of fit was assessed using the Kolmogorov-Smirnov test.

### Imaging data pre-processing

The spatial intensity inhomogeneities of the anatomical images were corrected based on proton density measurement information^37^ and applying a standard correction^38^ which uses low-order polynomials to model low-frequency variations. Then, the anatomical data were normalized to the AC-PC space^39^ and up-scaled to an isotropic resolution of 0.8 mm using a *sinc*-weighted interpolation.

Functional data for each participant were corrected for 3D body motion, aligning all subsequent runs to the functional run closest to the anatomical scans, and a temporal high-pass filtering (GLM-Fourier with two cycles sine/cosine per run, including linear trend removal) was applied. Furthermore, a distortion correction method^40^ based on opposite phase encoding of recorded functional volumes with distortions in opposite directions was applied. Finally, functional images were co-registered to the 3D anatomical data and resampled at the original resolution using a *sinc* interpolation.

### Imaging data analysis

A GLM was used to assess the functional activation patterns^41^. The predictors for conditions of interest were generated by convolving the activation blocks with the standard hemodynamic response function^42^. In the ambiguous runs, the activation blocks were defined separately for the two perceptual states as reported by the participants. For each stimulation protocol, all runs were analyzed together at the single subject level using GLM and including the motion parameters as confound predictors. The data were normalized according to z-transformation and corrected for serial correlations.

### Interhemispheric functional connectivity

We started by investigating the interhemispheric functional connectivity as the correlation between the right and left hMT+ activity time courses during the coherent and incoherent motion percepts per participant. The connectivity was estimated as the partial Spearman’s^43^ correlation between the right and left hMT+ ROIs conditioned on the time courses of a ROI with noisy signal (unrelated spherical ROI with 257 voxels defined on the white matter) using the *partialcorr* function of MATLAB Statistics Toolbox. The time courses of each ROI were extracted for each run and then the interhemispheric correlation was calculated from block to block along the time course depending on the perceived pattern of motion and taking into account the hemodynamic delay of six seconds. Only the blocks with a minimum duration of eight seconds were included. Also, blocks immediately after a baseline period were not included. On average we analyzed 28.56 ± 2.77 trials during which the participant perceived coherent motion (mean duration of 4.99 ± 0.33) and 32.11 ± 3.74 trials during which the participant perceived incoherent motion (mean duration of 5.04 ± 0.23). The correlation values per percept and per participant were assessed based on the mean of the correlation values calculated per blocks of each percept. Data from one participant was not included due to the low duration of incoherent percepts. Additionally, a control experiment for the interhemispheric functional connectivity fluctuation due to different motion orientation perception was performed. The hMT+ interhemispheric correlation during passive perceptual integration of vertical and horizontal motion was estimated based on the moving gratings used in localizer runs.

To further replicate correlation analysis per block (which has some limitations such as the need to discard blocks), we also estimated the interhemispheric functional connectivity based on the generalized psychophysiological interaction (gPPI) analyses^44^, which identifies a task-specific increase in the exchange of information between brain regions^45,46^. We applied the BrainVoyager’ PPI plugin per participant taking into account the motion parameters as confounds. Each ROI, right and left hMT+, was used as seed to extract the *beta* value of the contrast between the PPI predictors of each percept.

The correlation dynamics over perceptual switches was also analyzed. A centered sliding window of ten seconds was used to estimate the time courses of partial Spearman’s correlation during ambiguous stimulation. We then focused on the perceptual switch events from coherent to incoherent motion and vice-versa to analyze the group changes in interhemispheric correlation over time taking into account the hemodynamic delay of six seconds.

All correlation coefficients were converted to z scores using Fisher’s z transformation.

### Statistical analysis

Interhemispheric correlation groups’ differences were evaluated using a paired sample Wilcoxon signed-rank test (2-tailed). On the other hand, the probability of a higher interhemispheric psychophysiological interaction during the coherent percept than during the incoherent percept, at the group level, was estimated by a 2-tailed binomial test. Furthermore, a regression model was used to determine whether there was a significant relationship between the perceptual switches and the dynamics of interhemispheric functional connectivity.

## Supplementary Information

Supplementary Info

## Data Availability

The datasets generated during the current study are available from the corresponding author on reasonable request.
