# Supplementary material for: Tracking perceptual decision mechanisms through changes in interhemispheric functional connectivity in human visual cortex
Source: Sci Rep. 2019 Feb 4;9:1242. doi: 10.1038/s41598-018-37822-x (PMC6362201; doi:10.1038/s41598-018-37822-x)
Supplement: Supplementary file 1 — Supplementary Info [file 41598_2018_37822_MOESM1_ESM.docx]

**Tracking perceptual decision mechanisms through changes in interhemispheric functional connectivity in human visual cortex**

Teresa Sousa^1,2,3,4^, João V. Duarte^1,2,3^, Gabriel N. Costa^1,2,3^, Valentin G. Kemper^4^, Ricardo Martins^1,2,3^, Rainer Goebel^4,5^, Miguel Castelo-Branco^1,2,3 *^

[1] Coimbra Institute for Biomedical Imaging and Translational Research (CIBIT), University of Coimbra

[2] Institute of Nuclear Sciences Applied to Health (ICNAS), University of Coimbra

[3] Institute for Biomedical Imaging and Life Sciences (CNC.IBILI), Faculty of Medicine, University of Coimbra

[4] Department of Cognitive Neuroscience, Faculty of Psychology and Neuroscience, University of Maastricht

[5] Department of Neuroimaging and Neuromodeling, Netherlands Institute for Neuroscience, Royal Netherlands Academy of Arts and Sciences (KNAW)

**
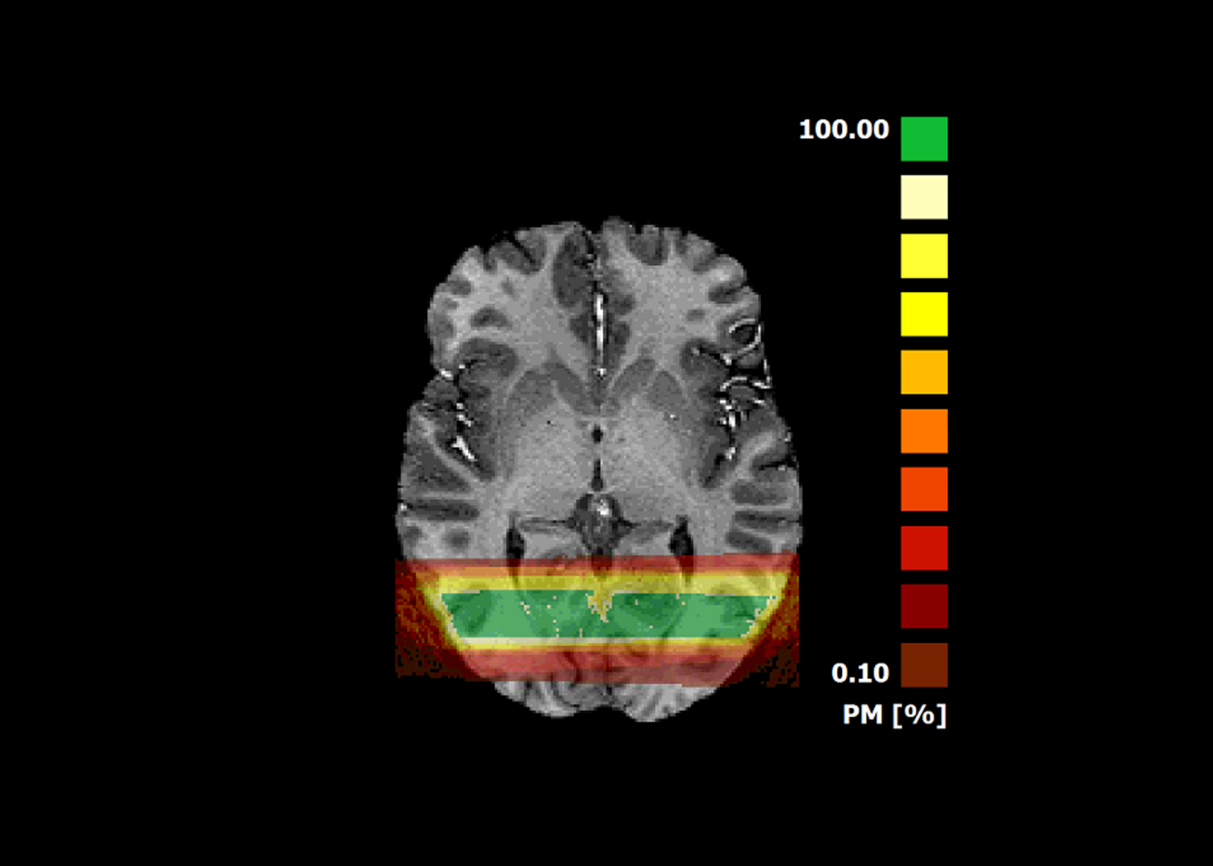
**

**Supplementary Figure S1.** Functional brain coverage of the acquired fMRI data. Color-coded probability map of the functional subspace that is covered by all participants’ data. The green color indicates those voxels where all participants contain functional coverage (see top square in color bar). To run this analysis the data were normalized into the Talairach space at the isotropic resolution of 1 mm.

**
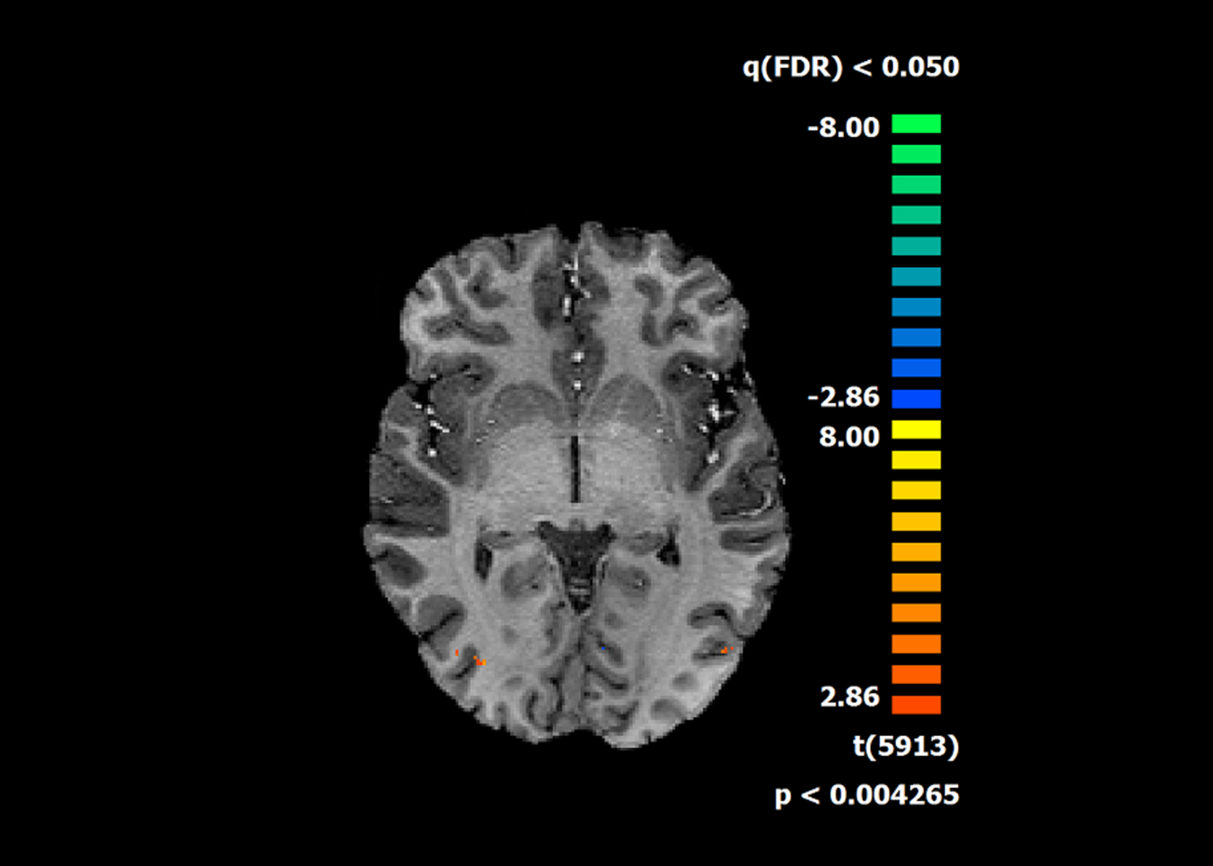
**

**Supplementary Figure S2.** Group analysis of the ambiguous stimulation data when contrasting incoherent and coherent percepts. Within the acquired slab, only hMT+ region shows significant differences between both percepts’ responses. To run this analysis the data were normalized into the Talairach space at the isotropic resolution of 1 mm.
